# Protective Effect of Hepcidin on Sepsis-Associated Acute Kidney Injury via Activating the Nrf2/GPX4 Signaling Pathway

**DOI:** 10.3390/cimb47090772

**Published:** 2025-09-18

**Authors:** Liang-Bo Guo, Shao-Sheng Wu, Feng Xu, Xin-Xing Chen, Heng Fan

**Affiliations:** Department of Intensive Care Unit, The First Affiliated Hospital of Ningbo University, Ningbo 315010, China; 2311140036@nbu.edu.cn (L.-B.G.); 2411140054@nbu.edu.cn (S.-S.W.); xufengnbu@163.com (F.X.); fyychenxinxing@nbu.edu.cn (X.-X.C.)

**Keywords:** hepcidin, sepsis, acute kidney injury, ferroptosis, Nrf2

## Abstract

Background: Hepcidin not only sustains systemic iron homeostasis but also functions as an antimicrobial peptide. During this study, we sought to analyze the ability of hepcidin to protect against sepsis-associated acute kidney injury (SAKI) and elucidated its underlying mechanisms in mediating ferroptotic pathways. Methods: A SAKI mouse model was created via cecal ligation and puncture (CLP), along with an LPS-induced Human Kidney-2 (HK-2) cell model, to study the protective mechanism of hepcidin against SAKI. Through the analysis of renal injury biomarkers and ferroptosis-related molecules, combined with quantitative detection of nuclear factor-erythroid 2-related factor-2 (Nrf2) nuclear translocation and glutathione peroxidase 4 (GPX4), a regulatory protein of ferroptosis, we uncovered the hepcidin-mediated mechanisms underlying ferroptosis in SAKI. Results: Hepcidin significantly attenuated renal function impairment in mice with SAKI and reduced the sepsis-driven increase in inflammatory mediators. As sepsis was associated with enhanced renal ferroptosis, hepcidin exerted a therapeutic effect by mitigating ferroptosis to a degree comparable with that of the ferroptosis inhibitor Ferrostatin-1 (Fer-1). Furthermore, hepcidin conferred renoprotective effects in SAKI by promoting the nuclear translocation of Nrf2, which in turn mediated the upregulation of the downstream anti-ferroptotic protein GPX4. Importantly, the Nrf2 inhibitor ML385 abrogated both the hepcidin-induced nuclear translocation of Nrf2 and the subsequent increase in GPX4 expression. Conclusions: Protective effects of hepcidin against SAKI are mediated by the Nrf2/GPX4 ferroptosis pathway, underscoring its therapeutic potential for SAKI.

## 1. Introduction

Sepsis, characterized by a dysregulated host response to infection leading to life-threatening organ dysfunction, is a life-threatening syndrome [1]. During the early phase of sepsis, the kidneys are often the first organs injured, resulting in decreased urine output and acute renal dysfunction, which are common characteristics of acute kidney injury (AKI). This form of acute kidney injury occurring in sepsis is referred to as sepsis-associated acute kidney injury (SAKI) [2]. Retrospective analysis of clinical data shows that approximately 47.1% of sepsis patients experience AKI [3]. In contrast to patients with other types of AKI, those with SAKI exhibit longer hospital stays and higher mortality rates [4]. Currently, the pathogenesis of SAKI remains unclear, with its pathological process being highly complex and clinical treatment options limited. This has rendered SAKI a significant medical problem threatening human life and health.

An iron-dependent mode of programmed cell death, ferroptosis features the abnormal overaccumulation of intracellular ferrous ions, excessive production of lipid peroxides, and dysfunction of the amino acid-based antioxidant system [5]. In sepsis, the core mechanism driving renal ferroptosis is the dysregulation of lipid peroxidation, whereby sepsis suppresses the renal amino acid–dependent antioxidant system—characterized by reduced GSH levels and impaired GPX4 activity—resulting in excessive lipid peroxide accumulation and ultimately triggering ferroptosis in renal tubular epithelial cells [6]. Studies have shown that in animal models of SAKI, significantly increased mitochondrial reactive oxygen species (ROS) induce ferroptosis in renal parenchymal cells, while the ferroptosis inhibitor Ferrostatin-1 effectively alleviates tissue injury in these animals [7]. Another study showed that melatonin alleviates SAKI by activating Nrf2-dependent HO-1 expression, thereby inhibiting ferroptosis of SAKI [8]. Conversely, by suppressing GPX4 expression in renal tubular epithelial cells, PGE2 induces ferroptosis, thereby further exacerbating renal injury [9]. In summary, these findings establish ferroptosis as a critical driver in the sepsis-induced renal deterioration. Although the specific mechanisms by which ferroptosis contributes to SAKI pathology remain incompletely understood, targeting the ferroptosis pathway is considered an effective strategy for alleviating SAKI and thus provides potential therapeutic targets for this condition.

Hepcidin is primarily synthesized in the liver and serves as an essential hormone regulating systemic iron homeostasis. It governs iron uptake and systemic distribution by binding to the iron exporter ferroportin (FPN) expressed on duodenal enterocytes and macrophages [10]. Studies have shown that hepcidin inhibits the ferroptosis process in septic acute lung injury by upregulating the expression of ferritin heavy chain (FTH) [11]. In lupus nephritis, hepcidin similarly alleviates renal tissue iron accumulation and inflammatory responses by promoting FTH expression [12]. In addition, in sepsis animal models, knockout of the hepcidin gene was found to lead to impaired phagocytic clearance capacity of neutrophils and macrophages against bacteria in septic mice, accompanied by a significant increase in blood bacterial load [13]. These studies demonstrate that modulation of hepcidin signaling may confer therapeutic benefits in SAKI. Nrf2, a critical nuclear antioxidant transcription factor, mediates its antioxidant effect by interacting with antioxidant response elements (ARE), a process that modulates the expression of antioxidant proteins and downstream signaling molecules [14]. Additionally, Gabapentin, isoorientin, and CBX7 all alleviate AKI by activating the Nrf2 pathway to inhibit oxidative stress [15,16,17]. These provide new insights into inhibiting ferroptosis in the treatment of SAKI. Through establishing in vivo cellular systems and ex vivo sepsis models, we deciphered the effects of hepcidin on SAKI and delineated its Nrf2/GPX4 signaling pathways in this study.

## 2. Materials and Methods

### 2.1. Experimental Animal

All male C57BL/6 mice (6–8 weeks old, weighing 20 ± 3 g) were acquired from the Laboratory Animal Center of Ningbo University (Ningbo, China). The experimental animals were kept in a specific pathogen-free (SPF) facility that sustains stable conditions at 22 ± 1 °C with a 12 h light/dark cycle and were provided with autoclaved acidified water and irradiated pellets. This study was conducted in strict accordance with the NIH Guide for the Care and Use of Laboratory Animals, and all animal procedures were approved by the Animal Ethics and Welfare Committee (AEWC) of Ningbo University.

The CLP method was applied to generate the murine model of SAKI; meanwhile, experimental mice that died within 24 h were excluded from the analysis because CLP-induced sepsis represents an acute inflammatory state [18]. Briefly, under isoflurane anesthesia, mice were subjected to a midline laparotomy to facilitate cecal exposure. Next, we circumferentially ligated the cecum at the midpoint between the cecal tip and the ileocecal valve using a 4-0 silk suture, created 1 microperforation with a 22G needle, and extruded a small amount of feces (approximately 1 mm) into the peritoneal cavity before abdominal closure. For fluid resuscitation, we subcutaneously injected mice with warm (37 °C) saline and guaranteed continuous availability of food and water after recovery from anesthesia. Postoperative analgesia was provided for all mice. Finally, we collected serum and renal tissues at 24 h post-CLP induction for further analysis. Based on inferences from existing literature and combined with Appendix A.2 preliminary experimental results, we administered hepcidin at a dose of 5 mg/kg via IP injection 24 h prior to CLP surgery, which was determined as the optimal concentration [19]. 60 mice were assigned to six groups via random numbers in simple randomization: Sham group (laparotomy without cecal ligation or puncture); Hamp group (sham operation + IP injection of hepcidin at the same volume as the treatment group); CLP group (cecal ligation and puncture alone); CLP + Hamp group (pretreatment with 5 mg/kg hepcidin 24 h before CLP); CLP + Hamp + ML385 group (pretreatment with 5 mg/kg hepcidin 24 h before CLP, followed by IP injection of 30 mg/kg ML385 2 h prior to CLP to inhibit Nrf2 activity) and CLP+Fer-1 group (IP injection of 5 mg/kg Fer-1 1 h prior to CLP). Exogenous hepcidin was synthesized by the Chinese pharmaceutical company QYAO-BIO (Shanghai, China); ML385 (HY-100523) and Fer-1 (HY-100579) were purchased from MCE (Monmouth Junction, NJ, USA).

### 2.2. Cell Lines

HK-2 cells were professionally procured from the Chinese Academy of Sciences Cell Bank, a certified cell repository. HK-2 cells were maintained under standard conditions: DMEM/F12 complete medium supplemented with 10% fetal bovine serum (C04001-500, Biological Industries, Kibbutz Beit HaEmek, Israel) and 1% penicillin-streptomycin, in a humidified 5% CO_2_ incubator at 37 °C. For a 24 h treatment period, HK-2 cells were exposed to LPS (L2630, Sigma-Aldrich, St. Louis, MO, USA) at 10 μg/mL, a condition determined by Appendix A.1 preliminary experimental results. Hepcidin (10 μg/mL) administered 24 h prior to LPS exposure attenuates inflammatory responses in HK-2 cells, based on Appendix A.1 preliminary experimental data. HK-2 cells were pretreated with ML385 (10 μM) for 1 h prior to LPS treatment, which inhibits Nrf-2 nuclear translocation.

### 2.3. Cell Viability

The CCK-8 kit (K1018, APExBIO, Houston, TX, USA) was utilized for the detection of cell viability. Approximately 4 × 10^3^ cells were seeded into individual wells of 96-well plates and allowed to adhere before treatment according to the experimental groups. After adding the prepared CCK-8 working solution, we shielded the cells from light and incubated them at 37 °C for 1 h to prevent photodegradation of the reagents, after which we used a multimode microplate reader (Spectra Max iD3, San Jose, CA, USA) to measure the absorbance at 450 nm.

### 2.4. ELISA Assay

Serum concentrations of the cytokines (TNF-α, IL-1β, IL-6), Neutrophil Gelatinase-Associated Lipocalin (NGAL), and Kidney Injury Molecule-1 (KIM-1) were quantified by ELISA with specific kits for each analyte: TNF-α (EK282, MULTI SCIENCES, Hangzhou, China), IL-1β (E-EL-M0037, Elabscience, Wuhan, China), NGAL (E-EL-M0828, Elabscience, Wuhan, China), KIM-1 (E-EL-M3039, Elabscience, Wuhan, China), and IL-6 (EK206, MULTI SCIENCES, Hangzhou, China). Additionally, levels of TNF-α (RK0030, ABclonal, Wuhan, China) and IL-6 (EK106, MULTI SCIENCES, Hangzhou, China) in cell culture supernatants were determined using the similar protocol as for serum samples. Detailed experimental procedures followed the manufacturers’ instructions provided with each kit.

### 2.5. Biochemical Analysis

In order to evaluate renal function, respective test kits were used to determine serum creatinine (C011-2-1) and BUN (C013-2-1) levels, both purchased from Nanjing Jiancheng Institute (Nanjing, China).

### 2.6. Ferrous Iron (Fe^2+^) Content in Renal Tissue

We measured Fe^2+^ levels in renal tissues of each group with the aid of a ferrous iron analysis kit (BC5415, Solarbio, Beijing, China). Approximately 100 mg of renal tissue was homogenized at −10 °C with the extraction buffer provided in the kit. When the homogenate was centrifuged at 4 °C for 10 min at 10,000× *g* in a pre-chilled centrifuge, we carefully collected the supernatant to avoid contamination for downstream analysis. After color development using the kit’s reagents, we used a multimode microplate reader (Spectra Max iD3, San Jose, USA) to measure sample absorbance at 593 nm.

### 2.7. GSH-Px Activity Assay

GSH-Px activity in renal tissues was assayed by virtue of a colorimetric kit (BC1195, Solarbio, Beijing, China) that relies on the reduction of 5,5′-dithiobis-(2-nitrobenzoic acid) (DTNB). A 20–30 mg aliquot of renal tissue was weighed and subjected to cryogenic grinding using a cryogenic grinder. The renal homogenate was centrifuged to harvest the supernatant, after which GSH-Px activity was assayed by adding the kit’s reaction reagents and measuring absorbance at 412 nm.

### 2.8. Detection of Lipid Peroxidation Product MDA and GSSH/GSSG Ratio

Levels of MDA, GSH, and GSSG in renal tissues were quantified with commercially purchased assay kits (Solarbio, Beijing, China), each containing pre-calibrated reagents for MDA (BC002), GSH (BC1175), and GSSG (BC1185).

### 2.9. Lipid ROS Assay

Intracellular ROS levels in HK-2 cells were assessed using a DCFH-DA fluorescent probe (2′,7′-dichlorodihydrofluorescein diacetate; G1706, Servicebio, Wuhan, China) following the manufacturer’s instructions. Briefly, after removing the culture medium and washing the cells twice with PBS, DCFH-DA was diluted in basal medium at a ratio of 1:1000 and incubated with the cells at 37 °C in the dark for 30 min. Following incubation, the cells were washed, detached, and collected by centrifugation (Thermo Fisher Scientific, Waltham, MA, USA) at 1200 rpm for 5 min. Fluorescence intensity was quantified using a BD Accuri C6 flow cytometer. In parallel, ROS generation was also visualized by fluorescence microscopy (DMI8, Leica, Wetzlar, Germany) under excitation/emission wavelengths of 485/530 nm.

### 2.10. Hematoxylin and Eosin Staining

Following fixation in 4% paraformaldehyde for 24 h, gradient ethanol dehydrated the renal tissues, xylene cleared them, and paraffin embedded them. Paraffin sections (5 μm thick) were stained with H&E, mounted using neutral gum, and examined under a 200× optical microscope (Nikon Eclipse E100, Nikon, Tokyo, Japan) to assess histological changes. Clear blue-stained cell nuclei and pink cytoplasmic structures were visualized. Tubular injury was defined as: swelling of renal tubular epithelial cells, loss of brush border, vacuolar degeneration, tubular dilation, necrosis, cast formation, and desquamation. The degree of tubular injury was evaluated using a semi-quantitative pathological scoring system [20]. Score 0: normal renal tissue; Score 1: <25% tubular injury; Score 2: 25–50% tubular injury; Score 3: 50–75% tubular injury; Score 4: 75–100% tubular injury.

### 2.11. Western Blot

Freshly isolated kidney specimens were lysed in RIPA buffer with protease inhibitors, homogenized on ice, centrifuged at 12,000× *g*, and normalized for protein concentration. The samples were denatured by boiling at 100 °C. SDS-PAGE separated equal amounts of renal tissue protein and subsequently transferred to PVDF membranes. After incubation in blocking buffer for 30 min, the PVDF membranes were incubated overnight at 4 °C with a mouse antibody against GPX4 (1:2000, 67763-1-Ig, Proteintech, Wuhan, China), a rabbit antibody against ACSL4 (1:10,000, 22401-1-AP, Proteintech, Wuhan, China), a rabbit antibody against Nrf2 (1:1000, A0674, ABclonal, Wuhan, China), a rabbit antibody against Lamin B1 (1:10,000, 12987-1-AP, Proteintech, Wuhan, China), and a mouse antibody against GAPDH (1:50,000, 60004-1-Ig, Proteintech, Wuhan, China). After washing the membranes three times with TBST, we incubated the PVDF membranes with Goat anti-rabbit (1:5000, SA00001-2, Proteintech, Wuhan, China) or Goat anti-mouse (1:5000, SA00001-1, Proteintech, Wuhan, China) IgG-HRP conjugates for 1 h. Chemiluminescent detection was performed using a Bio-Rad imaging system. Nuclear Protein Kit (PC204, Yazyme, Shanghai, China) separated Nrf2 nuclear protein from cytoplasmic fractions of renal tissues.

### 2.12. Immunohistochemistry

Mice renal tissues were immobilized via immersion in 4% paraformaldehyde buffered, processed into paraffin sections. First, after deparaffinizing and rehydrating the tissue sections, we incubated them overnight at 4 °C with a 1:1000 dilution of anti-Nrf2 primary antibody (GB113808, Servicebio, Wuhan, China). Then we incubated the sections with a corresponding secondary antibody at room temperature for 50 min. We used ImageJ (1.53e, NIH Co., Bethesda, MD, USA) to measure the integrated optical density (IOD) values as an indicator of Nrf2 expression level.

### 2.13. Apoptosis Rates

Apoptotic rates of HK-2 cells were quantified by Annexin V-APC/PI double staining using the Apoptosis Detection Kit (AT107, MultiSciences, Hangzhou, China) and analyzed with the BD FACSVia flow cytometer. HK-2 cells, including those in the culture supernatant, were pelleted by spinning at 1200 rpm for 5 min, suspended in 1 × Binding Buffer, double-stained with Annexin V-APC and PI. HK-2 cells were stained with Annexin V-APC and PI for 5 min at room temperature in the dark. Flow cytometry was used to analyze the apoptosis rate of HK-2 cells in each group.

### 2.14. RT-qPCR

Total RNA extraction from mouse kidney tissues and HK-2 cells was carried out with an RNA Extraction Kit (RN001, Yishen Biotechnology, Shanghai, China), followed by concentration assessment via NanoDrop spectrophotometry. cDNA was generated in 20 μL reaction systems using a Kit (RR036A, TaKaRa, Kusatsu, Japan) via reverse transcription, and target genes were amplified by qPCR using a Master Mix Kit (A6001, Promega, Madison, WI, USA). Transcript levels were quantified using the cycle threshold (CT) method, and relative mRNA expression levels were derived by applying the 2^−ΔΔCT^ method. All primer sequences were sourced from PrimerBank “https://pga.mgh.harvard.edu/primerbank/ (accessed on 1 September 2025)” and listed in Table 1.

### 2.15. Survival Analysis

After 15 mice in each group were subjected to distinct interventions as per the predefined experimental groups, survival rates were observed over a 72 h timeframe.

### 2.16. Statistical Analysis

All statistical data are presented as mean ± standard deviation (SD). Normality tests were first performed on data from each group. Nonparametric Kruskal–Wallis tests were used for non-normally distributed data. One-way ANOVA was used to compare differences among multiple groups, followed by Tukey’s post hoc tests to identify pairwise differences; Welch’s ANOVA was employed for data with unequal variances. Kaplan–Meier survival analysis was utilized to compare mortality rates among mice in different groups. Data plots were generated using GraphPad Prism software (Version 9.5; GraphPad, San Diego, CA, USA). Statistical significance was defined as *p* < 0.05.

## 3. Results

### 3.1. Hepcidin Ameliorates SAKI in Mice

**Figure 1:**
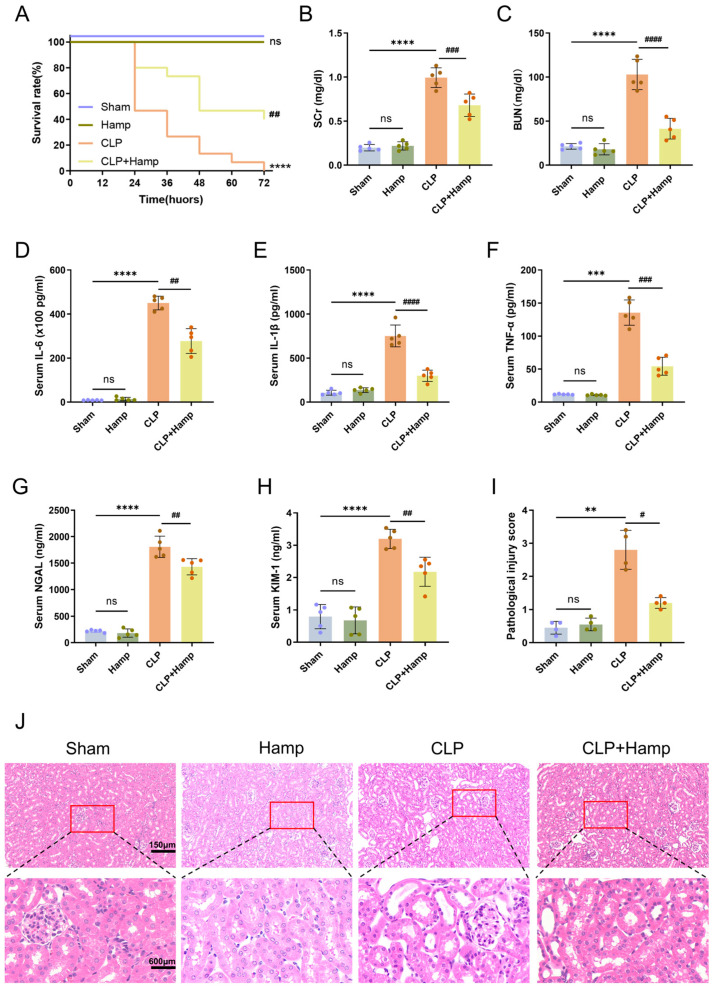
Hepcidin ameliorates SAKI in mice. (**A**) Survival rates of mice in each group over 72 h (*n* = 15 mice per group); (**B**) SCr level and (**C**) BUN level in different groups(*n* = 5 mice per group); (**D**) Serum IL-6 concentration, (**E**) serum IL-1β concentration, and (**F**) serum TNF-α concentration measured by ELISA (*n* = 5 mice per group); Serum kidney injury markers of NGAL (**G**) and KIM-1 (**H**) quantified by ELISA (*n* = 5 mice per group); (**I**) Semi-quantitative pathological scores of tubular injury(*n* = 4 mice per group); (**J**) Histopathological damage in kidney tissues of mice in each group under 200× light microscopy and with local magnification. Scale bar: 150 μm and 600 μm. Compared with the sham group, ns, no significant difference; ** *p* < 0.01, *** *p* < 0.001, **** *p* < 0.0001; Compared with the CLP group, ^#^ *p* < 0.05, ^##^ *p* < 0.01, ^###^ *p* < 0.001, ^####^ *p* < 0.0001. Hamp, hepcidin.

To evaluate the protective role of hepcidin against SAKI, we performed a survival analysis, which demonstrated that all CLP group mice died within 72 h, with the majority of deaths occurring between 24 and 48 h. In contrast, pretreatment with hepcidin reduced the mortality rate of SAKI mice to 40% (Figure 1A). We conducted biochemical assays for SCr and BUN, which revealed substantial elevations in the CLP group that were attenuated by hepcidin treatment (Figure 1B,C). In addition, we conducted ELISA assays to quantify serum levels of IL-6, IL-1β, and TNF-α, which were shown to exhibit a notable rise in the CLP group and were significantly reduced with hepcidin treatment (Figure 1D–F). To further determine the renoprotective effects of hepcidin, after being treated with hepcidin, the CLP mice exhibited substantially reduced renal injury markers (serum NGAL and KIM-1) levels (Figure 1G,H). Histopathological analysis by H&E staining showed that hepcidin treatment notably reduced tubular injury in CLP mice, characterized by decreased tubular epithelial cell swelling, lumen dilation, and epithelial detachment/necrosis. Semi-quantitative scoring revealed lower tubular injury scores in the hepcidin treatment group when the group was contrasted with the CLP group (Figure 1I,J). Collectively, these data demonstrate that hepcidin ameliorates kidney injury in the SAKI mice model.

### 3.2. Hepcidin Inhibits Ferroptosis in SAKI Mice

**Figure 2:**
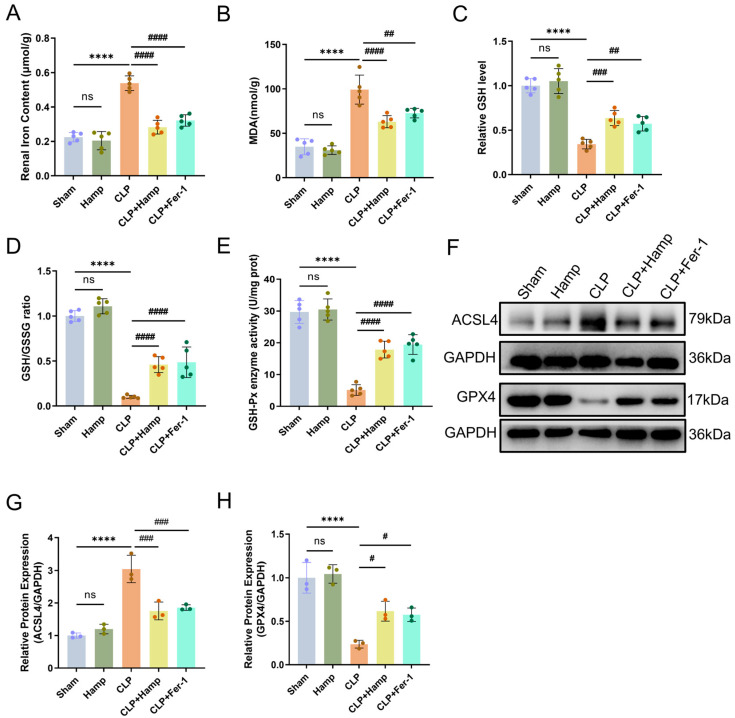
Hepcidin inhibits ferroptosis in SAKI mice. (**A**) The content of ferrous iron in renal tissues (*n* = 5 mice per group); (**B**) Renal tissue MDA levels (*n* = 5 mice per group); (**C**) GSH content in renal tissues (*n* = 5 mice per group); (**D**) GSH/GSSG ratio; (**E**) GSH-Px activity (*n* = 5 mice per group); (**F**) Ferroptosis-dependent protein expression changes in mice; Relative expression levels of ACSL4 (**G**) and GPX4 (**H**) in renal tissue (*n* = 3 mice per group); Compared with the sham group, ns, no significant difference; **** *p* < 0.0001; Compared with the CLP group, ^#^ *p* < 0.05, ^##^ *p* < 0.01, ^###^ *p* < 0.001, ^####^ *p* < 0.0001. Hamp, hepcidin; Fer-1, Ferrostatin-1.

Our study focused on assessing indicators related to ferroptosis and using the feroptosis inhibitor Fer-1 to confirm the effect of hepcidin on ferroptosis in SAKI. The result indicated that septic kidneys accumulated more Fe^2+^ than sham kidneys, but after hepcidin treatment, the accumulation of this iron was much less (Figure 2A). Hepcidin can reduce the sepsis-driven renal level of the lipid peroxidation marker MDA, and promote renal GSH production and GSSG conversion during sepsis, thereby reducing oxidative damage and rebalancing the disrupted GSH/GSSG ratio (Figure 2B–D). This is consistent with the levels of GSH-Px enzyme activity we observed in different groups of mice (Figure 2E). Western blot analysis of the ferroptosis related proteins ACSL4 and GPX4 showed that ACSL4 levels were significantly higher in septic kidneys than in the sham group, while GPX4 expression was markedly reduced; after hepcidin administration, ACSL4 levels decreased, while GPX4 levels increased (Figure 2F–H). Treatment with Fer-1 in SAKI had similar effects to hepcidin. In summary, hepcidin inhibited ferroptosis in SAKI mice.

### 3.3. Hepcidin Protects HK-2 Cells from the Damage That Is Induced by LPS

**Figure 3:**
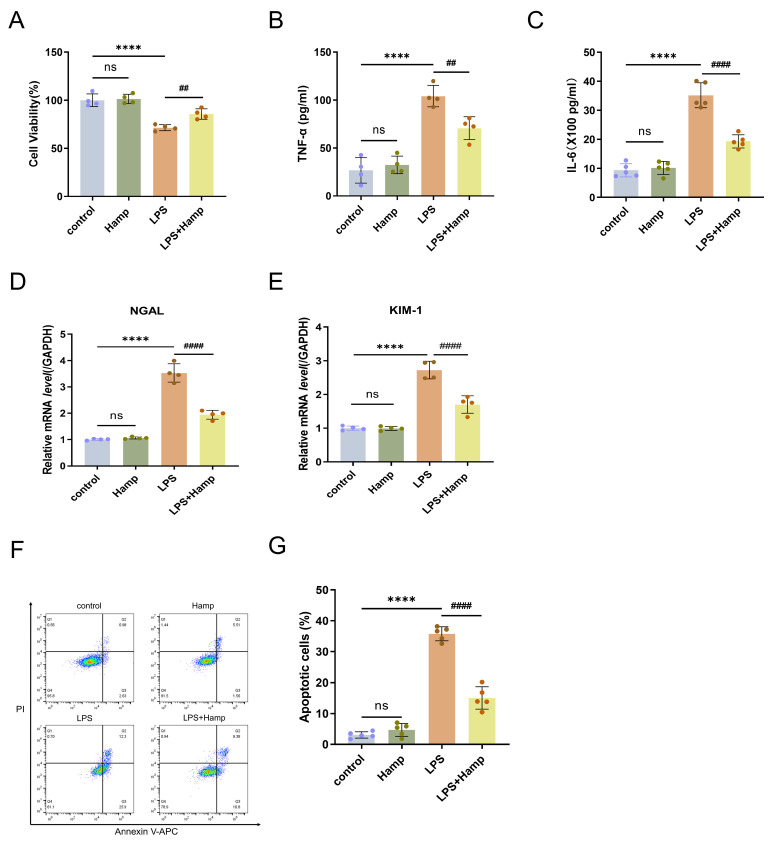
Hepcidin protects HK-2 cells from the damage that is induced by LPS. (**A**) HK-2 cell viability(*n* = 4 per group); TNF-α (**B**) and IL-6 (**C**) levels in HK-2 cell supernatants by ELISA (*n* = 4 or 5 per group); RT-qPCR analysis of NGAL (**D**) and KIM-1 (**E**) mRNA expression in HK-2 cells (*n* = 4 per group); (**F**) and (**G**) Analysis of the apoptosis rate of HK-2 cells by flow cytometry (*n* = 5 per group); Compared with the control group, ns, no significant difference; **** *p* < 0.0001; Compared with the LPS group, ^##^ *p* < 0.01, ^####^ *p* < 0.0001. Hamp, hepcidin.

Our experimental data showed that the toxicity of LPS significantly reduced the survival rate of HK-2 cells, while early treatment with hepcidin effectively reduced the damage caused by LPS and helped restore cell vitality (Figure 3A). LPS stimulation triggered an inflammatory response in HK-2 cells, which was manifested by an appreciable increase in TNF-α and IL-6 levels in cell culture supernatants; however, hepcidin pre-treatment suppressed the release of these two cytokines (Figure 3B,C). In addition, hepcidin reduced LPS-induced NGAL mRNA expression by 45%, and KIM-1 mRNA expression by 37.6%, indicating that hepcidin can reduce LPS damage to HK-2 cells (Figure 3D,E). Pretreatment with hepcidin for 24 h substantially reduced the apoptosis of HK-2 cells, reducing the apoptosis rate by 20.74% (Figure 3F,G). In summary, hepcidin both mitigates inflammatory damage caused by LPS to HK-2 cells and reduces the number of HK-2 cells that apoptose due to LPS.

### 3.4. Hepcidin Alleviates LPS-Induced Ferroptosis by Activating the Nuclear Translocation of Nrf2

**Figure 4:**
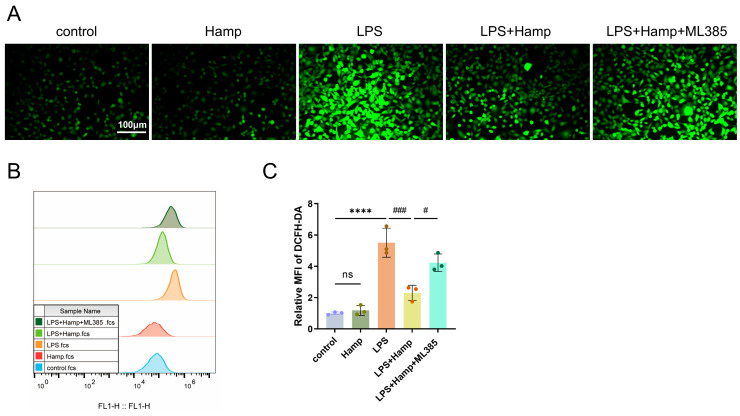
Hepcidin alleviates LPS-induced ferroptosis by activating the nuclear translocation of Nrf2. (**A**) ROS fluorescence images in HK-2 cells, Scale bar: 100 μm; (**B**) Flow cytometry analysis of ROS in HK-2 cells (*n* = 3 per group); (**C**) Flow cytometric analysis of relative fluorescence intensity of ROS (*n* = 3 per group). Compared with the control group, ns, no significant difference; **** *p* < 0.0001; Compared with the LPS+Hamp group, ^#^ *p* < 0.05, ^###^ *p* < 0.001. Hamp, hepcidin.

We assessed the effect of hepcidin on LPS-induced ROS accumulation, as ROS levels are a key indicator of cellular ferroptosis. Experimental results showed that LPS stimulation markedly elevated ROS production in HK-2 cells, with fluorescence intensity substantially stronger than that of the control group (Figure 4A). Importantly, hepcidin pretreatment significantly attenuated the LPS-induced increase in ROS. Flow cytometry confirmed these findings, showing that ROS levels in the LPS group were approximately fivefold higher than those in controls, whereas hepcidin reduced ROS accumulation to ~41.8% of that observed in the LPS group (Figure 4B,C). We treated samples with the Nrf2 inhibitor ML385 to block the translocation of Nrf2 to the nucleus. In LPS-treated HK-2 cells, the ROS-reducing effect of hepcidin was partially offset by inhibition of Nrf2 with ML385, demonstrating that hepcidin reduces LPS-induced ferroptosis by activating Nrf2 (Figure 4A–C).

### 3.5. Hepcidin Inhibits Ferroptosis in SAKI via Activation of Nuclear Translocation of Nrf2

**Figure 5:**
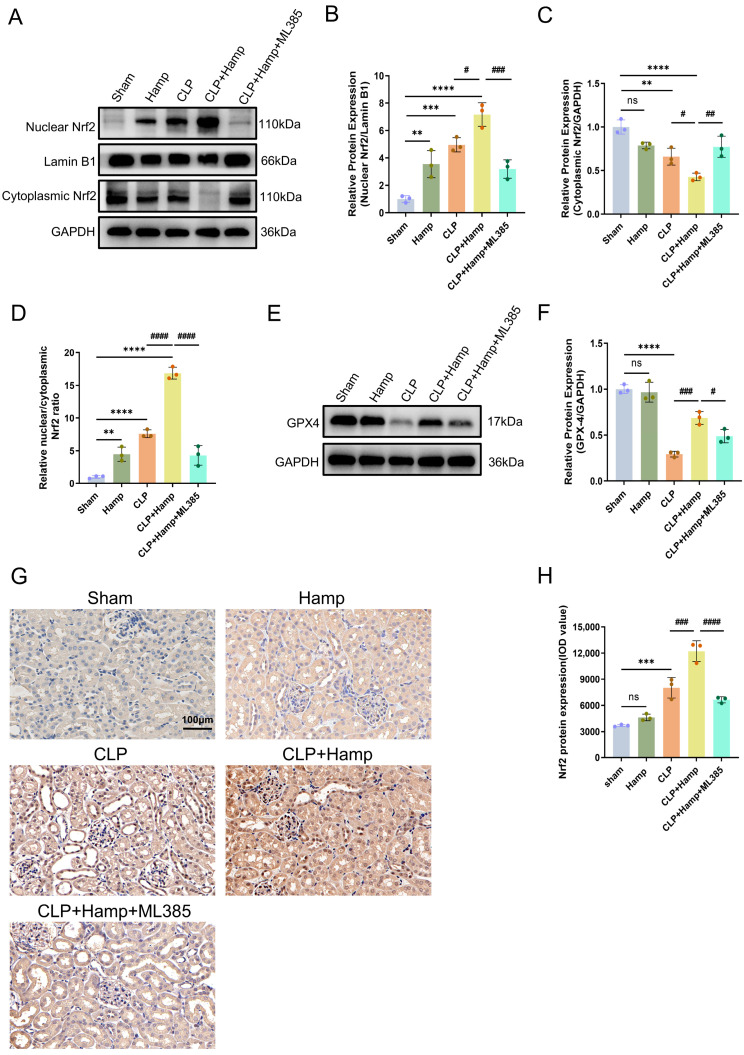
Hepcidin inhibits ferroptosis in SAKI via activation of nuclear translocation of Nrf2. (**A**) Nuclear Nrf2 and cytoplasmic Nrf2 protein bands in renal tissue by WB; (**B**) Relative Nrf2 levels (Lamin B1) in renal nuclear fractions (*n* = 3 mice per group); (**C**) Relative Nrf2 levels (GAPDH) in renal cytoplasmic fractions(*n* = 3 mice per group); (**D**) Nuclear-to-cytoplasmic Nrf2 ratio in renal tissue (*n* = 3 per group); (**E**) GPX4 protein bands in renal tissue by Western blot; (**F**) Relative GPX4 protein levels by Western blot (GAPDH) in renal tissue lysates (*n* = 3 mice per group); (**G**) Nrf2 protein expression by IHC; (**H**) Quantitative analysis of Nrf2 protein IOD values by IHC (*n* = 3 mice per group). Compared with the sham group, ns, no significant difference; ** *p* < 0.01, *** *p* < 0.001, **** *p* < 0.0001; Compared with the CLP+Hamp group, ^#^ *p* < 0.05, ^##^ *p* < 0.01, ^###^ *p* < 0.001, ^####^ *p* < 0.0001. Hamp, hepcidin.

As shown by the experimental results, in comparison with the sham group, CLP-induced septic mice exhibited increased nuclear Nrf2 expression in the kidney, accompanied by a concurrent decrease in cytoplasmic Nrf2. Notably, hepcidin treatment in septic mice further enhanced nuclear Nrf2 levels while significantly reducing its cytoplasmic expression, resulting in a nuclear-to-cytoplasmic Nrf2 ratio in the hepcidin treatment group that was markedly higher than those in both the sham and CLP groups (Figure 5A–D). However, adding ML385(Nfr2 inhibitor) to hepcidin treatment weakened the effect: nuclear Nrf2 declined and cytoplasmic Nrf2 rose compared to hepcidin alone, with the nuclear/cytoplasmic ratio approaching that of the septic mice (Figure 5A–D). Furthermore, Western blot showed that GPX4 protein levels were suppressed in CLP-induced mice compared to sham mice but prominently increased by pre-hepcidin, and this trend was reversed by ML385, leading to decreased GPX4 expression (Figure 5E,F). The immunohistochemical results of Nrf2 indicating that CLP-induced SAKI promoted the expression of Nrf2 in mice kidneys, and treatment with hepcidin further enhanced this accumulation of Nrf2 (Figure 5G,H). In summary, hepcidin inhibits ferroptosis in SAKI by activating nuclear translocation of Nrf2 to upregulate downstream GPX4 expression, thereby alleviating renal injury.

## 4. Discussion

Immunomodulation, antimicrobial therapy, and inhibition of oxidative stress have been recognized as key research priorities in the treatment of SAKI [21,22,23]. However, despite these therapeutic efforts, a subset of patients inevitably progresses to irreversible renal failure, resulting in markedly poor prognosis [24]. This clinical challenge largely stems from the lack of agents that directly and specifically target the pathological mechanisms underlying SAKI. Consequently, the development of mechanism-based targeted therapies has become a central focus in the field. In this context, our study demonstrates that hepcidin exerts a protective effect against SAKI by attenuating ferroptosis. Mechanically, hepcidin activates the antioxidant transcription factor Nrf2, thereby promoting the expression of the downstream anti-ferroptotic protein GPX4. This regulatory axis effectively suppresses lipid peroxidation in renal tissue and contributes to the mitigation of SAKI.

Previous investigations indicate that sepsis is frequently accompanied by dysregulation of hepcidin, most commonly manifesting as increased expression [25]. Mechanistically, this dysregulation is largely driven by the host inflammatory response: proinflammatory cytokines such as IL-6 activate transcriptional pathways—most notably the JAK/STAT3 signaling cascade—that enhance HAMP gene transcription [26]. Meanwhile, exogenous hepcidin supplementation at 100 μg/mouse alleviates pathology in a sepsis-associated lung injury mouse model, suggesting it holds therapeutic value for mitigating sepsis [11]. Our findings similarly demonstrate that hepcidin alleviates inflammatory responses in both septic mice and LPS-induced HK-2 cells, thereby exerting a protective effect against AKI. This beneficial action stems from hepcidin’s capacity to suppress the excessive production of IL-6 and TNF-α in LPS-stimulated HK-2 cells, alongside its ability to inhibit the nuclear factor-κB (NF-κB)/p53 signaling pathway ultimately resulting in a decreased apoptosis rate in HK-2 cells [27,28]. This is consistent with our observation that hepcidin treatment reduces LPS-induced apoptosis in HK-2 cells. Based on these results, we found that hepcidin, besides improving renal histopathological manifestations and alleviating tubular injury, also significantly reduced mortality in SAKI mice, indicating that hepcidin holds great potential for ameliorating SAKI. Furthermore, hepcidin can significantly reduce the release of proinflammatory factors, indicating that it possesses favorable immunomodulatory functions. However, this study did not conduct in-depth investigations to clarify its impact on macrophages.

Further investigations have demonstrated that inhibiting renal ferroptosis effectively alleviates SAKI [29]. Given that ferrous iron overload is a critical step in ferroptosis [5], our data demonstrate that hepcidin suppresses the Fe^2+^-driven Fenton reaction triggered by iron overload in SAKI, which in turn inhibits the subsequent elevation of MDA. Hepcidin intervention reduced both ferrous iron accumulation and MDA production. Accumulating evidence indicates that ROS and lipid peroxides reduce GSH, increase GSSG, decrease the GSH/GSSG ratio, and reduce GSH-Px activity, leading to redox imbalance and ferroptosis [30]. In line with previous observations, our experimental data have shown that SAKI mice exhibited reduced renal GSH, increased GSSG, a decreased GSH/GSSG ratio, and diminished GSH-Px activity, all of which were reversed by hepcidin treatment. Earlier studies have also demonstrated that ACSL4 promotes lipid peroxide formation, whereas GPX4 degrades lipid peroxides using oxidized GSH, and inhibiting ACSL4 or overexpressing GPX4 suppresses ferroptosis [31,32]. Meanwhile, hepcidin exhibits similarities to the ferroptosis inhibitor Fer-1, a ferroptosis inhibitor investigated in previous studies on sepsis-induced acute kidney injury (SAKI), in that both exert their effects by inhibiting ferroptosis [33].

In sepsis, renal tubular epithelial cells that absorb endotoxins suffer oxidative stress-mediated damage [34]. The underlying mechanism may involve the activation of NOX4 (NADPH Oxidase 4) in SAKI, which promotes reactive ROS production and subsequent renal dysfunction [35]. This finding explains why LPS stimulation results in increased ROS production in HK-2 cells, as observed in our flow cytometry experiments and the ROS fluorescence intensity assay detected by DCFH-DA staining, whereas hepcidin suppresses ROS accumulation, thus mitigating cellular oxidative damage. When ML385 inhibited Nrf2 nuclear translocation, it negated the effect of hepcidin in alleviating LPS-induced excessive ROS production. This implies that hepcidin’s anti-ferroptotic function relies on Nrf2 nuclear translocation to upregulate downstream anti-ferroptotic proteins, as validated by earlier studies [36,37].

Under oxidative stress, Nrf2, functioning as a pivotal antioxidant transcription factor, dissociates from the Kelch-like ECH-associated protein 1 (KEAP1) repressor complex and exerts antioxidant effects [38]. Further studies have shown that Nrf2 transcriptionally activates downstream GPX4 and HO-1, two key antioxidant enzymes, to counteract oxidative stress [39]. Given the critical role of Nrf2 in maintaining redox homeostasis, we confirmed the effects of hepcidin on Nrf2 in SAKI through Western blot. Western blot validation, the experimental findings provided robust evidence that hepcidin potently enhanced the nuclear translocation of Nrf2 in renal tubular epithelial cells. Through IHC analysis and Western blot validation provided robust evidence that hepcidin potently enhanced the nuclear translocation of Nrf2 in renal tissue. This finding aligns with pre-studies showing that Nrf2 activation upregulates downstream antioxidant proteins, thereby mitigating SAKI animal models [40]. GPX4, an essential mediator of ferroptosis, catalyzes the conversion of GSH to GSSG, scavenges lipid peroxides, and thus inhibits cellular ferroptosis [41]. Previous studies have validated that targeting the Nrf2/GPX4 axis effectively mitigates renal dysfunction in sepsis, a finding that supports our conclusion [42,43,44]. Our results showed that hepcidin significantly increased GPX4 expression in renal tissues of SAKI mice. To further determine whether hepcidin acts through the Nrf2/GPX4 axis, we used the specific Nrf2 inhibitor ML385 to block its activity and found that GPX4 expression was subsequently decreased, suggesting that the regulation of GPX4 by hepcidin is dependent on Nrf2 activation. Furthermore, in a septic setting, IκBα phosphorylation activates NF-κB to induce excessive proinflammatory cytokines and inflammatory damage, while activated Nrf2 (after nuclear translocation) transcribes antioxidant genes and inhibits IκBα phosphorylation to block NF-κB, thereby suppressing inflammation [45]; hepcidin also promotes Nrf2 nuclear translocation and reduces serum proinflammatory cytokines in septic mice, suggesting it may block NF-κB. To assess whether hepcidin employs other Nrf2-controlled pathways, in Appendix A.3 RT-PCR experiments, we observed that hepcidin could upregulate the LPS-induced reduced mRNA expression of FTH1, HO-1, and SLC7A11, while exerting no effect on FSP1 mRNA. These results indicate that hepcidin can activate the expression of downstream anti-ferroptotic proteins FTH1, HO-1, and SLC7A11 by enhancing Nrf2 nuclear translocation, suggesting that hepcidin has several ways to regulate ferroptosis. As long-term use of this molecule theoretically carries the risk of inducing iron overload, our current study—which only focuses on short-term intervention—has limitations when it comes to assessing long-term safety. To address these gaps, we have outlined our future research directions. Specifically, we intend to perform long-term animal experiments to further investigate the protective role of hepcidin in SAKI. In addition, immunofluorescence analysis in HK-2 cells will be employed to more precisely determine the subcellular localization of Nrf2 and to provide clearer and more direct evidence of the effects of hepcidin on LPS-treated HK-2 cells. In summary, hepcidin enhances nuclear translocation of Nrf2, thereby upregulating downstream GPX4 expression to inhibit ferroptosis and alleviate SAKI.

## 5. Conclusions

**Figure 6:**
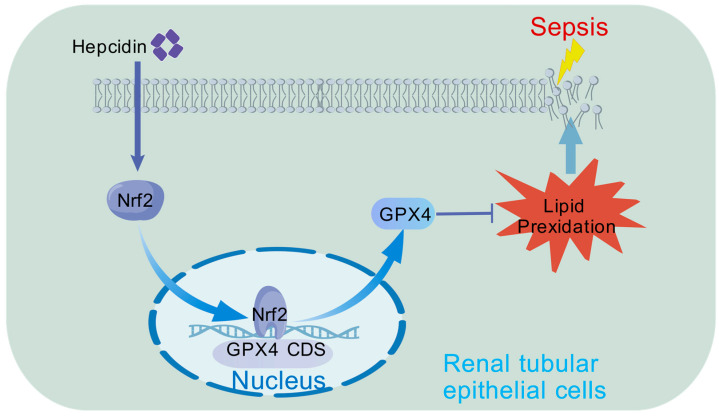
Hepcidin treatment may alleviate sepsis-associated acute kidney injury through activating the Nrf2/GPX4 pathway to suppress ferroptosis in SAKI.

In conclusion, our study revealed that ferroptosis plays a pivotal role in the pathogenesis of SAKI (Figure 6). Targeting this process may therefore represent a promising therapeutic strategy for mitigating disease progression. Mechanistically, hepcidin promotes Nrf2 nuclear translocation, leading to enhanced GPX4 expression and ultimately contributing to the attenuation of SAKI.

## Figures and Tables

**Table 1:** RT-qPCR Primer.

Primer	Sequences 5’-3’	PrimerBank ID
NGAL For NGAL Rev	GAAGTGTGACTACTGGATCAGGA ACCACTCGGACGAGGTAACT	108936956c3
KIM-1 For KIM-1 Rev	TGGCAGATTCTGTAGCTGGTT AGAGAACATGAGCCTCTATTCCA	290560749c1
HO-1 For HO-1 Rev	AAGACTGCGTTCCTGCTCAAC AAAGCCCTACAGCAACTGTCG	298676487c1
FTH1 For FTH1 Rev	TCCTACGTTTACCTGTCCATGT GTTTGTGCAGTTCCAGTAGTGA	4503795a2
FSP1 For FSP1 Rev	GATGAGCAACTTGGACAGCAA CTGGGCTGCTTATCTGGGAAG	4506765a1
SLC7A11 For SLC7A11 Rev	TCTCCAAAGGAGGTTACCTGC AGACTCCCCTCAGTAAAGTGAC	80861465c1
GAPDH For GAPDH Rev	GGAGCGAGATCCCTCCAAAAT GGCTGTTGTCATACTTCTCATGG	378404907c1

**Scheme A1:**
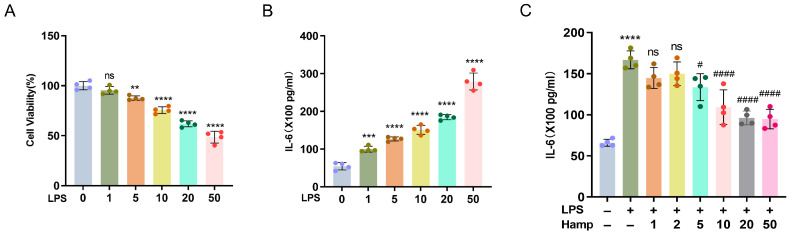
Optimal LPS Stimulation and Hepcidin Intervention Concentrations in HK-2 Cells. (**A**) effects of LPS (μg/mL) on the viability of HK-2 cells; (**B**) IL-6 levels in the supernatant of HK-2 cells at LPS (μg/mL); (**C**) effects of hepcidin (μg/mL) on LPS-triggered IL-6 in HK-2 cell supernatant. *n* = 4 per group; compared with the control group, ns indicates no significant difference, ** *p* < 0.01, *** *p* < 0.001, **** *p* < 0.0001; compared with the LPS group, ns indicates no significant difference, ^#^ *p* < 0.05, ^####^ *p* < 0.0001. Hamp, hepcidin.

**Scheme A2:**
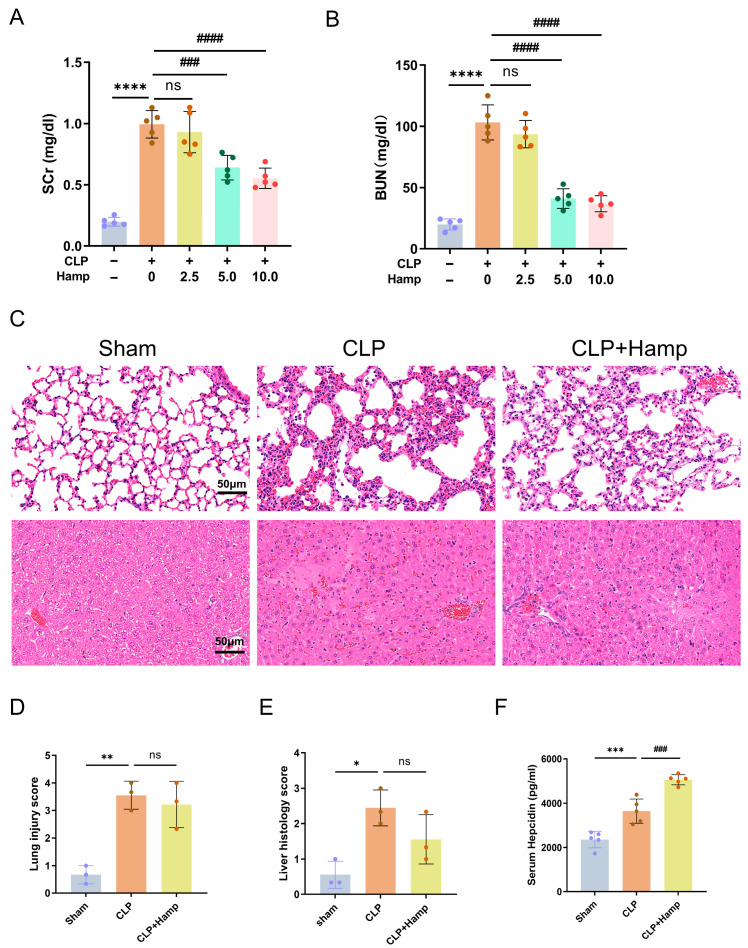
Hepcidin’s effects on sepsis-induced liver injury, lung injury, concentration-dependent renal function, and serum hepcidin level. (**A**) effects of hepcidin (mg/kg) on the SCr (*n* = 5 per group); (**B**) effects of hepcidin (mg/kg) on the serum BUN(*n* = 5 per group); (**C**) Histopathological damage in lung and liver tissues of mice in each group under 200× light microscopy and with local magnification. Scale bar: 50 μm, *n* = 3 per group; (**D**) and (**E**) Semi-quantitative pathological scores of lung and liver injury(*n* = 3 mice per group); (**F**) Serum hepcidin level(*n* = 5 per group); compared with the sham group, ns indicates no significant difference, * *p* < 0.05, ** *p* < 0.01, *** *p* < 0.001, **** *p* < 0.0001; compared with the CLP group, ns indicates no significant difference, ^###^ *p* < 0.001, ^####^ *p* < 0.0001. Hamp, hepcidin.

**Scheme A3:**
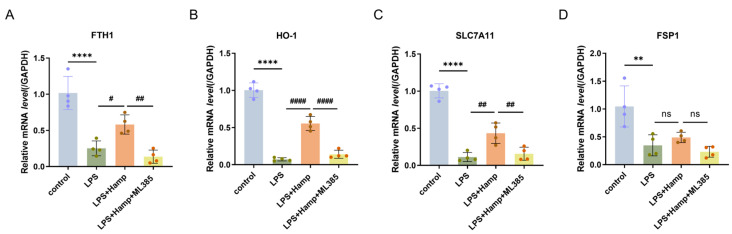
Hepcidin’s modulatory role in Nrf2 downstream target genes HO-1, SLC7A11, FSP1, and FTH1. FTH1 (**A**), HO-1 (**B**), SLC7A11 (**C**) and FSP1 (**D**) mRNA levels in HK-2 cells (*n* = 4 per group); compared with the control group, ns indicates no significant difference, ** *p* < 0.01, **** *p* < 0.0001; compared with the LPS+Hamp group, ns indicates no significant difference, ^#^ *p* < 0.05, ^##^ *p* < 0.01, ^####^ *p* < 0.0001. Hamp, hepcidin.

## Data Availability

The data that support the findings of this study are available on request from the corresponding author.

## Appendix A

### Appendix A.1

To determine optimal LPS (for stimulating HK-2 cells) and hepcidin (for intervention) concentrations, we evaluated HK-2 viability at different LPS concentrations and IL-6 levels in supernatants, then examined how varying hepcidin concentrations regulated LPS-triggered IL-6 secretion in these cells. The results indicated that cell viability decreased to 75.55% in 10 μg/mL LPS-treated cells, and this value was notably different from the cell viability of the control (Scheme A1A). Moreover, when LPS concentration reached or exceeded 5 μg/mL, IL-6 levels in the supernatant of HK-2 cells were significantly elevated in comparison to the control (Scheme A1B). Lastly, treatment with hepcidin on LPS-stimulated HK-2 cells resulted in a mild reduction in IL-6 levels at 5 μg/mL hepcidin, whereas a prominent decrease was observed at 10 μg/mL (Scheme A1C). In summary, 10 μg/mL was determined as the optimal therapeutic concentration of hepcidin, and this concentration was also identified as the optimal dose for LPS induction in the present study.

### Appendix A.2

Experimental results showed that hepcidin at a dose of 5 mg/kg had a protective effect on SAKI, but there was no more benefit when increased to 10 mg/kg (Scheme A2A,B). However, examination of liver and lung tissues by HE staining showed that hepcidin did not have a significant effect on sepsis-induced liver damage, such as patchy necrosis, inflammatory cell infiltration, or local lobular structural disorders, nor did it improve lung damage, including alveolar destruction/fusion, septal thickening, inflammatory cell infiltration, or intrapulmonary vascular damage (Scheme A2C–E). In addition, the serum hepcidin concentration in septic mice (3640.0 ± 550.4 pg/mL) was significantly higher than that in the sham group (2357.0 ± 374.5 pg/mL), and the level increased even more after the administration of exogenous hepcidin (5058.0 ± 229.2 pg/mL) (Scheme A2F).

### Appendix A.3

RT-qPCR was used to investigate the effect of hepcidin on the expression of Nrf2 downstream target genes in HK-2 cells. LPS significantly reduced the mRNA levels of HO-1, SLC7A11, FSP1 and FTH1 compared to the untreated control group. In contrast, hepcidin treatment restored the transcription levels of HO-1, SLC7A11 and FTH1, but exerted minimal effect on FSP1 (Scheme A3A–D). Moreover, co-treatment with the selective Nrf2 inhibitor ML385 abolished the hepcidin-mediated upregulation of HO-1, SLC7A11 and FTH1 was blocked (Scheme A3A–D).

## References

[1] Singer M., Deutschman C.S., Seymour C.W., Shankar-Hari M., Annane D., Bauer M., Bellomo R., Bernard G.R., Chiche J.D., Coopersmith C.M. (2016). The Third International Consensus Definitions for Sepsis and Septic Shock (Sepsis-3). JAMA.

[2] Zarbock A., Nadim M.K., Pickkers P., Gomez H., Bell S., Joannidis M., Kashani K., Koyner J.L., Pannu N., Meersch M. (2023). Sepsis-associated acute kidney injury: Consensus report of the 28th Acute Disease Quality Initiative workgroup. Nat. Rev. Nephrol..

[3] Xu X., Nie S., Liu Z., Chen C., Xu G., Zha Y., Qian J., Liu B., Han S., Xu A. (2015). Epidemiology and Clinical Correlates of AKI in Chinese Hospitalized Adults. Clin. J. Am. Soc. Nephrol. CJASN.

[4] Bagshaw S.M., Uchino S., Bellomo R., Morimatsu H., Morgera S., Schetz M., Tan I., Bouman C., Macedo E., Gibney N. (2007). Septic acute kidney injury in critically ill patients: Clinical characteristics and outcomes. Clin. J. Am. Soc. Nephrol. CJASN.

[5] Dixon S.J., Lemberg K.M., Lamprecht M.R., Skouta R., Zaitsev E.M., Gleason C.E., Patel D.N., Bauer A.J., Cantley A.M., Yang W.S. (2012). Ferroptosis: An iron-dependent form of nonapoptotic cell death. Cell.

[6] Duo H., Yang Y., Luo J., Cao Y., Liu Q., Zhang J., Du S., You J., Zhang G., Ye Q. (2025). Modulatory role of radioprotective 105 in mitigating oxidative stress and ferroptosis via the HO-1/SLC7A11/GPX4 axis in sepsis-mediated renal injury. Cell Death Discov..

[7] Liang N.N., Zhao Y., Guo Y.Y., Zhang Z.H., Gao L., Yu D.X., Xu D.X., Xu S. (2022). Mitochondria-derived reactive oxygen species are involved in renal cell ferroptosis during lipopolysaccharide-induced acute kidney injury. Int. Immunopharmacol..

[8] Qiu W., An S., Wang T., Li J., Yu B., Zeng Z., Chen Z., Lin B., Lin X., Gao Y. (2022). Melatonin suppresses ferroptosis via activation of the Nrf2/HO-1 signaling pathway in the mouse model of sepsis-induced acute kidney injury. Int. Immunopharmacol..

[9] Liu Y., Zhou L., Lv C., Liu L., Miao S., Xu Y., Li K., Zhao Y., Zhao J. (2023). PGE2 pathway mediates oxidative stress-induced ferroptosis in renal tubular epithelial cells. FEBS J..

[10] Nemeth E., Tuttle M.S., Powelson J., Vaughn M.B., Donovan A., Ward D.M., Ganz T., Kaplan J. (2004). Hepcidin regulates cellular iron efflux by binding to ferroportin and inducing its internalization. Science.

[11] Jiao Y., Yong C., Zhang R., Qi D., Wang D. (2022). Hepcidin Alleviates LPS-Induced ARDS by Regulating the Ferritin-Mediated Suppression of Ferroptosis. Shock.

[12] Scindia Y., Wlazlo E., Ghias E., Cechova S., Loi V., Leeds J., Ledesma J., Helen C., Swaminathan S. (2020). Modulation of iron homeostasis with hepcidin ameliorates spontaneous murine lupus nephritis. Kidney Int..

[13] Stefanova D., Raychev A., Deville J., Humphries R., Campeau S., Ruchala P., Nemeth E., Ganz T., Bulut Y. (2018). Hepcidin Protects against Lethal Escherichia coli Sepsis in Mice Inoculated with Isolates from Septic Patients. Infect. Immun..

[14] Yamamoto M., Kensler T.W., Motohashi H. (2018). The KEAP1-NRF2 System: A Thiol-Based Sensor-Effector Apparatus for Maintaining Redox Homeostasis. Physiol. Rev..

[15] Abdelnaser M., Alaaeldin R., Attya M.E., Fathy M. (2024). Modulating Nrf-2/HO-1, apoptosis and oxidative stress signaling pathways by gabapentin ameliorates sepsis-induced acute kidney injury. Naunyn-Schmiedeberg’s Arch. Pharmacol..

[16] Fan X., Wei W., Huang J., Liu X., Ci X. (2020). Isoorientin Attenuates Cisplatin-Induced Nephrotoxicity Through the Inhibition of Oxidative Stress and Apoptosis via Activating the SIRT1/SIRT6/Nrf-2 Pathway. Front. Pharmacol..

[17] Zhang Y., Zhang J.J., Liu X.H., Wang L. (2020). CBX7 suppression prevents ischemia-reperfusion injury-induced endoplasmic reticulum stress through the Nrf-2/HO-1 pathway. Am. J. Physiol. Ren. Physiol..

[18] Rittirsch D., Huber-Lang M.S., Flierl M.A., Ward P.A. (2009). Immunodesign of experimental sepsis by cecal ligation and puncture. Nat. Protoc..

[19] Rivera S., Nemeth E., Gabayan V., Lopez M.A., Farshidi D., Ganz T. (2005). Synthetic hepcidin causes rapid dose-dependent hypoferremia and is concentrated in ferroportin-containing organs. Blood.

[20] Fan H., Le J.W., Zhu J.H. (2020). Protective Effect of N-Acetylcysteine Pretreatment on Acute Kidney Injury in Septic Rats. J. Surg. Res..

[21] Zhang L., Rao J., Liu X., Wang X., Wang C., Fu S., Xiao J. (2023). Attenuation of Sepsis-Induced Acute Kidney Injury by Exogenous H_2_S via Inhibition of Ferroptosis. Molecules.

[22] Wu Y., Wang L., Li Y., Cao Y., Wang M., Deng Z., Kang H. (2024). Immunotherapy in the context of sepsis-induced immunological dysregulation. Front. Immunol..

[23] Tong S.Y.C., Venkatesh B., McCreary E.K. (2023). Acute Kidney Injury with Empirical Antibiotics for Sepsis. JAMA.

[24] Flannery A.H., Li X., Delozier N.L., Toto R.D., Moe O.W., Yee J., Neyra J.A. (2021). Sepsis-Associated Acute Kidney Disease and Long-term Kidney Outcomes. Kidney Med..

[25] Moro H., Bamba Y., Nagano K., Hakamata M., Ogata H., Shibata S., Cho H., Aoki N., Sato M., Ohshima Y. (2023). Dynamics of iron metabolism in patients with bloodstream infections: A time-course clinical study. Sci. Rep..

[26] Wang J., Sun Q., Wang G., Wang H., Liu H. (2022). The effects of blunt snout bream (*Megalobrama amblycephala*) IL-6 trans-signaling on immunity and iron metabolism via JAK/STAT3 pathway. Dev. Comp. Immunol..

[27] De Domenico I., Zhang T.Y., Koening C.L., Branch R.W., London N., Lo E., Daynes R.A., Kushner J.P., Li D., Ward D.M. (2010). Hepcidin mediates transcriptional changes that modulate acute cytokine-induced inflammatory responses in mice. J. Clin. Investig..

[28] Chu W., Sun X., Yan Y. (2025). Study on the regulation of renal tubular cell apoptosis by SIRT1/NF-κB signaling pathway in septic acute kidney injury. Ren. Fail..

[29] Wang Y., Lv W., Ma X., Diao R., Luo X., Shen Q., Xu M., Yin M., Jin Y. (2024). NDUFS3 alleviates oxidative stress and ferroptosis in sepsis induced acute kidney injury through AMPK pathway. Int. Immunopharmacol..

[30] Ursini F., Maiorino M. (2020). Lipid peroxidation and ferroptosis: The role of GSH and GPx4. Free Radic. Biol. Med..

[31] Liu Y., Bao D., She H., Zhang Z., Shao S., Wu Z., Wu Y., Li Q., Wang L., Li T. (2024). Role of Hippo/ACSL4 axis in ferroptosis-induced pericyte loss and vascular dysfunction in sepsis. Redox Biol..

[32] Chu L.K., Cao X., Wan L., Diao Q., Zhu Y., Kan Y., Ye L.L., Mao Y.M., Dong X.Q., Xiong Q.W. (2023). Autophagy of OTUD5 destabilizes GPX4 to confer ferroptosis-dependent kidney injury. Nat. Commun..

[33] Qiongyue Z., Xin Y., Meng P., Sulin M., Yanlin W., Xinyi L., Xuemin S. (2022). Post-treatment with Irisin Attenuates Acute Kidney Injury in Sepsis Mice Through Anti-Ferroptosis via the SIRT1/Nrf2 Pathway. Front. Pharmacol..

[34] Kalakeche R., Hato T., Rhodes G., Dunn K.W., El-Achkar T.M., Plotkin Z., Sandoval R.M., Dagher P.C. (2011). Endotoxin uptake by S1 proximal tubular segment causes oxidative stress in the downstream S2 segment. J. Am. Soc. Nephrol. JASN.

[35] Li J., Wang L., Wang B., Zhang Z., Jiang L., Qin Z., Zhao Y., Su B. (2023). NOX4 is a potential therapeutic target in septic acute kidney injury by inhibiting mitochondrial dysfunction and inflammation. Theranostics.

[36] Lou Y., Shi H., Sha N., Li F., Gu X., Lin H. (2025). Ursodeoxycholic acid protects against sepsis-induced acute kidney injury by activating Nrf2/HO-1 and inhibiting NF-κB pathway. BMC Nephrol..

[37] Xu Z., Zhang M., Wang W., Zhou S., Yu M., Qiu X., Jiang S., Wang X., Tang C., Li S. (2023). Dihydromyricetin attenuates cisplatin-induced acute kidney injury by reducing oxidative stress, inflammation and ferroptosis. Toxicol. Appl. Pharmacol..

[38] Itoh K., Wakabayashi N., Katoh Y., Ishii T., Igarashi K., Engel J.D., Yamamoto M. (1999). Keap1 represses nuclear activation of antioxidant responsive elements by Nrf2 through binding to the amino-terminal Neh2 domain. Genes Dev..

[39] Xiao P., Huang H., Zhao H., Liu R., Sun Z., Liu Y., Chen N., Zhang Z. (2024). Edaravone dexborneol protects against cerebral ischemia/reperfusion-induced blood-brain barrier damage by inhibiting ferroptosis via activation of nrf-2/HO-1/GPX4 signaling. Free Radic. Biol. Med..

[40] Xie R.C., Zhang J.C., Huang T., Lin X.M., Wang Y.T., Zhang L.F., Hong X.Y., Lin X.F., Zheng H.J., Zhou K.L. (2025). Complement C5aR blockade attenuates LPS-induced acute kidney injury by regulating ferroptosis via nuclear factor-erythroid 2-related factor 2 signaling in mice. Free Radic. Biol. Med..

[41] Zhu L., Chen D., Zhu Y., Pan T., Xia D., Cai T., Lin H., Lin J., Jin X., Wu F. (2021). GPX4-Regulated Ferroptosis Mediates S100-Induced Experimental Autoimmune Hepatitis Associated with the Nrf2/HO-1 Signaling Pathway. Oxidative Med. Cell. Longev..

[42] Shen J., Chen S., Li X., Wu L., Mao X., Jiang J., Zhu D. (2024). Salidroside Mediated the Nrf2/GPX4 Pathway to Attenuates Ferroptosis in Parkinson’s Disease. Neurochem. Res..

[43] Liu J.X., Yang C., Liu Z.J., Su H.Y., Zhang W.H., Pan Q., Liu H.F. (2020). Protection of procyanidin B2 on mitochondrial dynamics in sepsis associated acute kidney injury via promoting Nrf2 nuclear translocation. Aging.

[44] Huang J., Zhao Y., Luo X., Luo Y., Ji J., Li J., Lai J., Liu Z., Chen Y., Lin Y. (2024). Dexmedetomidine inhibits ferroptosis and attenuates sepsis-induced acute kidney injury via activating the Nrf2/SLC7A11/FSP1/CoQ10 pathway. Redox Rep. Commun. Free Radic. Res..

[45] Zhang Z., Chen C., Zhou J., Li C., Du X., Hou H., Cao M., Yu D., Zhang J., Gu J. (2025). Carboxymethyl Poria cocos polysaccharides protect against septic kidney injury by regulating the Nrf2-NF-κB signaling pathway. Int. J. Biol. Macromol..

